# Reviving hope: ghrelin’s role in post-cardiac arrest coma management

**DOI:** 10.1097/MS9.0000000000002989

**Published:** 2025-02-06

**Authors:** Maria Qadri, Izere Salomon

**Affiliations:** a Jinnah Sindh Medical University, Karachi, Pakistan; b College of Medicine and Health Sciences, University of Rwanda, Kigali, Rwanda

*Dear Editor*,

After resuscitation from out-of-hospital cardiac arrest, approximately 80% of patients admitted to the ICU are in a comatose state. Of these comatose patients, approximately two-thirds eventually succumb to hypoxic-ischemic brain injury^[^1^]^. Post-cardiac arrest coma mainly stems from a post-cardiac arrest brain injury, which occurs due to the initial lack of blood flow during the cardiac arrest and the subsequent reperfusion injury following resuscitation. This can result in neuronal damage, cerebral edema, disturbance of cerebral blood flow, and impairments in cognition, motor, and sensory functions, and death^[^2,3^]^. The current treatment for post-cardiac arrest coma involves lowering body temperature to decrease metabolic rate and relieve reperfusion injury, as well as using sedatives that lower metabolic rate and inhibit the release of excitatory neurotransmitters^[^2,3^]^. Due to the large incidence of post-cardiac arrest coma, despite the use of these available regimens, it becomes imperative to look for a safer and more efficacious solution.

Ghrelin, also known as the hunger hormone, is a naturally produced hormone that plays a crucial role in signaling the availability of nutrients from the gastrointestinal tract to the brain. In a different study, it was shown that acylated ghrelin provides protection against neurotoxicity induced by 6-hydroxydopamine (6-OHDA) in a model of Parkinson’s disease. Ghrelin’s protective effects were linked to its capacity to enhance dysfunction in autophagic flux and facilitate the fusion of autophagosomes with lysosomes^[^4^]^.

The recently published GRECO trial, a phase 2, double-blind, placebo-controlled, multicentre, randomized clinical trial that included 160 patients, assessed the occurrence of coma within 12 hours of cardiac arrest^[^5^]^. The results of the trial reported that the use of acyl-ghrelin for treating post-cardiac arrest coma found a tendency toward better neurological outcomes^[^5^]^. In the intervention group, mortality was lower (37%) compared to the control group (51%). The safety profile of ghrelin was also promising, as there were fewer severe and common adverse events^[^5^]^. Furthermore, in an observational study that was published in Resuscitation, 50 individuals who had gone into cardiac arrest had their ghrelin levels tested. Higher ghrelin levels during the first 72 hours following cardiac arrest were linked to better neurological outcomes at 6 months, as determined by the Cerebral Performance Category Scale. This study, in line with the GRECO trial, shows that ghrelin might have neuroprotective properties and be a useful biomarker for estimating the possibility of neurological recovery following cardiac arrest^[^1^]^.

In Asia, post-cardiac arrest coma is still a major worry, as research shows that around 50% of individuals who survive cardiac arrest remain in a coma state for over 72 hours^[^6^]^. The trial and the evidence from previous research suggest that ghrelin has the potential to be used as a treatment to improve neurological outcomes in patients who are in a coma state following cardiac arrest. More studies and clinical trials are necessary to validate and build upon these results to pave the way for possible advancements in the care of post-cardiac arrest coma patients.

## Data Availability

Not applicable.
